# Clinical features of community acquired adenovirus pneumonia during the 2011 community outbreak in Southern Taiwan: role of host immune response

**DOI:** 10.1186/s12879-017-2272-5

**Published:** 2017-03-07

**Authors:** Ching-Fen Shen, Shih-Min Wang, Tzong-Shiann Ho, Ching-Chuan Liu

**Affiliations:** 1 Department of Pediatrics, National Cheng Kung University Hospital, College of Medicine, National Cheng Kung University, 138, Sheng Li Road, North Dist., Tainan, 70403 Taiwan; 2 Department of Emergency Medicine, National Cheng Kung University Hospital, College of Medicine, National Cheng Kung University, Tainan, Taiwan; 3 Center of Infectious Disease and Signaling Research, National Cheng Kung University Hospital, College of Medicine, National Cheng Kung University, Tainan, Taiwan; 4 Center for Infection Control, National Cheng Kung University Hospital, College of Medicine, National Cheng Kung University, Tainan, Taiwan

**Keywords:** Human adenovirus, Pneumonia, Pleural effusion, Immune response, T cells

## Abstract

**Background:**

Human adenovirus 7 (HAdV-7) was responsible for a significant number of fatalities during the 2011 community outbreak in Taiwan. The mechanisms underlying the pathogenesis of severe adenovirus infections in non-immunocompromised individuals remain unclear. Adenovirus pneumonia was associated with pleural effusion in a number of patients from the 2011 outbreak suggesting that similar to bacterial pneumonia, patients diagnosed with adenovirus pneumonia who have pleural effusion are more severely and systemically infected, and may have a more protracted disease course. We hypothesized that the host immunological response determines the severity of adenoviral infection.

**Methods:**

This retrospective case series study included patients diagnosed with severe lower respiratory tract infections at the National Cheng Kung University Hospital in southern Taiwan between December 2010 and October 2011. The main inclusion criteria were 1) presence of multifocal patchy infiltrates, lobar consolidation or reticular interstitial opacities in chest X-rays, and 2) presence of adenovirus isolated from respiratory specimens. All patients had adenovirus isolated from respiratory specimens, and were negative for other viruses. Pleural effusion was confirmed in all patients using chest echography. Clinical features and laboratory data were compared in patients with (*n* = 12) and without (*n* = 15) parapneumonic effusion.

**Results:**

Presence of parapneumonic effusion was significantly associated with a longer febrile duration, more complicated clinical management, and a greater risk of extrapulmonary involvement, notably hepatitis. Patients without pleural effusion had significantly higher numbers of WBCs, platelets, and absolute segment cell counts (ASCs) compared to patients with pleural effusion (all *p* < 0.05). Patients without pleural effusion had significantly higher counts of CD4+, CD8+, and CD20+ T cells (all *p* < 0.05) compared to patients with pleural effusion.

**Conclusion:**

Our data indicated that presence of parapneumonic effusion in adenoviral pneumonia was associated with longer febrile duration, more complicated clinical management, a greater risk of hepatitis, and suppression of host cellular immunity. Further prospective, large-scale studies are needed to validate our results.

## Background

Human adenoviruses (HAdVs) have been linked to a number of respiratory, gastrointestinal, ocular, genitourinary and neurologic diseases [1]. There are currently 60 serotypes of HAdVs identified, which are grouped into 7 species based on their morphologic, immunogenic and genomic properties [2, 3]. HAdV infections have been linked to mild upper respiratory tract infections such as pharyngitis or coryza, as well as to more severe lower respiratory tract infections (LRTIs) such as bronchiolitis, croup and pneumonia [4]. Approximately one-fifth of all HAdV infections were shown to be caused by HAdV-7, which is associated with serious LRTIs [5]. Although treatment of severe adenovirus infection is mainly supportive, antiviral agents such as acyclovir, ganciclovir, ribavirin, and cidofovir have been investigated for non-immunocompromised individuals [6, 7]. Therapeutic strategies such as extracorporeal membrane oxygenation (ECMO) with or without high frequency oscillatory ventilation (HFOV) have also been evaluated in patients with severe disease [8, 9].

Children, immunocompromised individuals, and people in crowded environments such as military recruits and hospitalized or institutionalized individuals have been shown to have a higher susceptibility to HAdV infections, especially HAdV-7 [10–13]. HAdVs 1, 2 and 5 are usually implicated in endemic or sporadic cases, while HAdVs 7 and to a lesser extent HAdV-3 have been isolated from epidemics of adenovirus LRTIs in healthy children [11, 14]. HAdV-11 and HAdV-7 were the most frequently identified pathogens in a 2011 study of community-acquired pneumonia in Beijing, China [15]. HAdV-7 was also reported as the primary pathogen in an outbreak of severe LRTI among infants in Shaanxi Province, China in 2009, and Singapore in 2012 [5, 16]. A number of adenovirus outbreaks involving HAdV-7, HAdV-3, and HAdV-4 have been reported from Taiwan between 1999 and 2005 [17, 18]. The largest reported community outbreak which occurred in Taiwan in 2011 involved co-circulating HAdV-3 and HAdV-7 [19]. Although the 2011 outbreak was associated with HAdV-3 as well as HAdV-7, patients infected with HAdV-7 had a higher fatality rate [20]. HAdV-7 isolated from the 2011 outbreak was shown to have the highest homology with HAdV-7d and HAdV-7d2, strains which had not been seen in Taiwan prior to 2011 [19].

Cellular immunity plays an important role in viral infections, and severe adenovirus infections are commonly seen in immunocompromised individuals. The mechanisms underlying the pathogenesis of severe adenovirus infections in non-immunocompromised individuals remain unclear. Production of chemokines and cytokines in response to HAdV-7 infection was shown to damage lung tissue, and result in recruitment of inflammatory cells such as neutrophils, monocytes and lymphocytes [21, 22]. Interestingly, a recent report reviewed 19 studies which evaluated immunocompetent patients with adenovirus pneumonia and showed that more than half the patients were lymphopenic [23]. A recent retrospective study also showed that non-immunocompromised patients with severe adenovirus pneumonia had lower hemoglobin levels, leucopenia, thrombocytopenia and elevated liver enzymes, suggesting a distinctive mechanism of immunopathogenesis which may contribute to the severity of the infection [7].

We hypothesized that the host immunological response determines the severity of adenoviral infection. Additionally, we observed that adenovirus pneumonia was associated with pleural effusion in a number of patients from the 2011 outbreak. We hypothesized that similar to bacterial pneumonia, patients diagnosed with adenovirus pneumonia who have pleural effusion are more severely and systemically infected, and may have a more protracted disease course. In this retrospective case series study, we evaluated the clinical manifestations of adenoviral pneumonia in a convenience sample of 27 patients from the 2011 outbreak in South Taiwan and compared clinical features and laboratory data between patients with or without parapneumonic effusion.

## Methods

### Materials

Blood cultures were done using the BACTEC Fx System (BD Biosciences, USA). Urine pneumococcal antigen tests were done with the BinaxNOW® *Streptococcus pneumoniae* Antigen Card (Alere Ltd, Stockport, UK), and urine Legionella antigen tests were done with the BinaxNOW *Legionella* Urinary Antigen Card (Alere Ltd, Stockport, UK). The Cryptococcus antigen test was done with the CALAS® Cryptococcal Antigen latex agglutination system (Meridian Bioscience, Inc, Cincinnati, Ohio, USA). Mycoplasma antibodies were detected using the Serodia Myco II gelatin particle agglutination kit (Fujirebio, Tokyo, Japan). The influenza rapid test was performed with the Directigen Flu A + B test (Directigen; BD Diagnostic Systems, Sparks, MD.) CDC-validated FDA-approved real time RT-PCR assays were used to detect human influenza virus (CDC influenza division, USA). The respiratory syncytial virus (RSV) antigen test was a direct fluorescent-antibody assay (DFA) by Diagnostic Hybrids Inc. (DHI; Athens, OH).

### Patients

This retrospective, single-center, observational case series included a total of 27 patients who presented with severe adenovirus LRTIs at the National Cheng Kung University (NCKU) Hospital in southern Taiwan between December 2010 and October 2011. Inclusion criteria were 1) presence of multifocal patchy infiltrates, lobar consolidation or reticular interstitial opacities in chest X-rays, and 2) presence of adenovirus isolated from respiratory specimens. Pleural effusion and diagnosis of adenoviral pneumonia was confirmed in all study patients using chest echography, which is an ultrasound technique. Chest echography at the NCKU hospital is mainly performed by senior pediatric residents with at least three years of pediatric training. The results are subsequently confirmed by the attending pediatrician and another pediatric chest specialist.

All throat swabs were examined for the presence of influenza virus using PCR assays, and for the presence of RSV antigen using a direct fluorescent-antibody assay. All specimens were negative for influenza and RSV. Co-infections were excluded using blood cultures, sputum cultures, pleural effusion cultures, the urine pneumococcal antigen test, the urine Legionella antigen test, the influenza rapid test, the RSV rapid test, PCR assay for influenza and the Cryptococcus and Mycoplasma tests. Data for presence of Mycoplasma antibodies were available for 21 patients (19 patients were negative and 2 patients were positive for Mycoplasma). Data for Legionella antibodies were only available for 3 patients, all of whom were negative for Legionella. Only two cases with significantly elevated levels of mycoplasma antibody presented with simple adenovirus pneumonia. Since our results would not have changed even if we had excluded these two cases, we retained these two cases in the adenovirus pneumonia without pleural effusion group. All other viral cultures only showed the presence of adenovirus. Although the antibiotic regimen varied from patient to patient, most patients were on ampicillin/sulbactam. All patient data were recorded soon after hospitalization. It is important to note that at the NCKU Hospital, all pediatric patients except those with walking/atypical pneumonia (caused by mycoplasma) are hospitalized for evaluation and treatment, and their medical data are accessible using administrative hospital data. This decreases the likelihood of selection bias within this sample of hospitalized patients.

This study sample was a convenience sample. Patients with adenovirus pneumonia usually have prolonged fever and severe respiratory symptoms, which increases the likelihood of seeking medical care. Most patients were referred by their local practitioners if their fever did not subside on the 3rd or 4th day. Our hospital is one of the biggest and largest pediatric tertiary referral centers in southern Taiwan, and even when the study cases are sampled by convenience, they are likely to closely reflect the population of patients hospitalized with severe adenoviral infection.

This study was approved by the Institutional Review Board of the National Cheng Kung University (NCKU) Hospital.

### Statistical analysis

Continuous data for the two groups of patients (patients with or without pleural effusion) were represented as either (i) mean ± SD and compared using the two-sample *t*-test if the data followed normal distribution or (ii) median (IQR: 1st, 3rd quartiles) and compared using the Mann-Whitney U test if the data didn’t follow normal distribution. Categorical data were represented as n (%) in the two groups of patients and compared using the Fisher’s exact test. All statistical assessments were two-tailed and *p* values <0.05 were considered significant. All statistical analyses were carried out with IBM SPSS statistical software version 22 for Windows (IBM Corp., New York, USA).

## Results

**Fig. 1:**
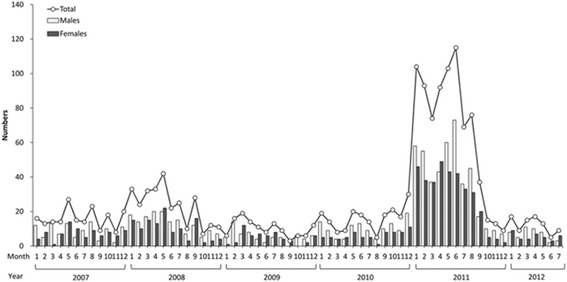
Number of patients per month from whom adenoviruses were isolated at the NCKU hospital during 2007–2012/July. The number above the circle means the total number of patients for the given month

This case series included a total of 27 patients who were admitted to the NCKU hospital with adenovirus pneumonia during the 2011 epidemic. Figure 1 represents the number of patients per month from whom adenoviruses were isolated at the NCKU hospital between 2007 and 2012.

In our present study, 12 study patients (44.4%) were classified into the pleural effusion group and 15 patients (55.6%) were classified into the non-pleural effusion group. The clinical characteristics of the two groups are summarized in Table 1. There were no significant differences in clinical characteristics between patients with and without pleural effusion (all *p* > 0.05). The mean age of the patients was 10 years (SD = 12.1, range: 1 to 42 years).

**Table 1:** Clinical characteristics of patients with adenovirus pneumonia admitted to the NCKU hospital during the 2011 epidemic (*N* = 27)

Variables	Total (*n* = 27)	Adenoviral pneumonia without pleural effusion (*n* = 15)	Adenoviral pneumonia with pleural effusion (*n* = 12)	*P* value
Age, years	4.1 (2.6 , 16.0)	3.7 (2.0 , 5.1)	5.6 (3.0 , 19.4)	0.213
Sex, males (%)	17 (63.0)	8 (53.3)	9 (75.0)	0.424
Underlying disease	2 (7.4)	1 (6.7)	1 (8.3)	1.000
Fever days at admission	6 (4 , 7)	5 (3 , 7)	7 (5 , 9.5)	0.092
Radiological findings				
Multifocal infiltrates	12 (44.4)	7 (46.7)	5 (41.7)	1.000
Lobar pneumonia	13 (48.1)	6 (40.0)	7 (58.3)	0.449
Pneumonitis	2 (7.4)	2 (13.3)	0 (0.0)	0.487
Chest tube replacement	6 (22.2)	0 (0.0)	6 (50.0)	NA
Genotypes				0.303
3S	15 (55.6)	10 (66.7)	5 (41.7)	
7S	9 (33.3)	3 (20)	6 (50)	
Lost	3 (11.1)	2 (13.3)	1 (8.3)	

Data were expressed as median (IQR: 1st, 3rd quartiles) for continuous variables and *n* (%) for categorical ones

Differences between patients with and without pleural effusion were compared using the Mann-Whitney U test for continuous variables and Fisher’s exact test for categorical ones

There were no significant differences between patients with and without pleural effusion

*NA* not assessed

The study population comprised 17 males (63%). Two patients (7.4%) had underlying disease. Study patients had an average of 6 days of fever at admission (SD = 3, range: 1 to 14 days). The radiological findings showed that 12 patients (44.4%) had multifocal infiltrates, 13 patients (48.1%) had lobar pneumonia, and 2 patients (7.4%) had pneumonitis. Additionally, 6 patients (22.2%) had chest tube replacement. Genotype analysis showed that 15 patients (55.6%) had genotype 3 s, whereas 9 patients (33.3%) had genotype 7 s. Genotype data were not available for 3 patients.

Clinical findings in the two study groups are presented in Table 2. Patients without pleural effusion had a significantly lower number of febrile days (7 days vs. 10.5 days; *p* = 0.022), a significantly lower number of hospital days (5 days vs. 11 days; *p* = 0.002), a significantly lower ICU admission rate (9 out of 10 patients admitted to the ICU had pleural effusion; *p* = 0.001), and a significantly lower rate of oxygen requirement (10 out of 13 patients who required oxygen had pleural effusion; *p* = 0.002) compared to patients without pleural effusion.

**Table 2:** Clinical findings in patients with adenovirus pneumonia admitted to the NCKU hospital during the 2011 epidemic based on the presence or absence of pleural effusions

Variables	Total (*n* = 27)	Adenoviral pneumonia without pleural effusion (*n* = 15)	Adenoviral pneumonia with pleural effusion (*n* = 12)	*P* value
Clinical symptoms other than respiratory tracts				
Conjunctivitis	1 (3.7)	0 (0.0)	1 (8.3)	NA
Gastroenterocolitis	11 (40.7)	7 (46.7)	4 (33.3)	0.696
Hepatitis	8 (29.6)	0 (0.0)	8 (66.7)	NA
Encephalitis	1 (3.7)	0 (0.0)	1 (8.3)	NA
Total febrile days	7 (6 , 11)	7 (5 , 9)	10.5 (7 , 12.8)	0.022^*^
Hospitalization days	7 (5 , 12)	5 (4 , 8)	11 (7.5 , 14.5)	0.002^*^
Duration for fever after admission, days	2 (0 , 4)	1 (0 , 4)	3.5 (0.3 , 4.8)	0.277
Patients with ICU admission	10 (40.7)	1 (6.7)	9 (75.0)	0.001
ICU hospitalization days^a^	6.0 (3.8 , 10.25)	9 (NA)	6 (3.5 , 11.5)	NA
Patients with ventilator	3 (11.9)	1 (6.7)	2 (16.7)	0.569
Patients with oxygen requirement	13 (48.1)	3 (20.0)	10 (83.3)	0.002^*^
Days of oxygen requirement	5 (4 , 12)	5 (4 , 14)	5 (4 , 13.3)	0.931

Data were expressed as median (IQR: 1st, 3rd quartiles) for continuous variables and *n* (%) for categorical ones

Difference between patients with and without pleural effusion were compared using the Mann-Whitney U test for continuous variables and Fisher’s exact test for categorical ones

*NA* not assessed

^*^ *p* < 0.05, indicates a significant difference between patients with and without pleural effusion

^a^Of 10 patients requiring ICU hospitalization, one patient had adenovirus pneumonia without pleural effusion, and nine patients had adenovirus pneumonia with pleural effusion

A comparison of laboratory findings in the two study groups is presented in Table 3. Our data showed that patients without pleural effusion had significantly higher WBC counts, platelet counts, and ASC compared to patients with pleural effusion (all *p* < 0.05).

**Table 3:** Laboratory examinations of patients with adenovirus pneumonia based on the presence or absence of pleural effusion (*N* = 27)

Variables	Total (*n* = 27)	Adenoviral pneumonia without pleural effusion (*n* = 15)	Adenoviral pneumonia with pleural effusion (*n* = 12)	*P* value
WBC count, cells/mm^3^	9674 ± 3427	10926.7 ± 3043.6	8108.3 ± 3340.8	0.031*
Platelet, 10^3^ cells/mm^3^	236 ± 108	285.1 ± 108.2	175.8 ± 75.5	0.007*
ANC, cells/mm^3^	7451 ± 3173	8738.4 ± 2879.7	6291.7 ± 3255.9	0.090
ASC, cells/ mm^3^	6039 ± 2674	7083.4 ± 2370.8	4733.9 ± 2531.2	0.020*
ABC, cells/ mm^3^	1397 ± 912	1268.1 ± 903.8	1557.8 ± 934.7	0.423
ALC, cells/ mm^3^	1444 ± 1064	1677.3 ± 1204.2	1152.2 ± 814.9	0.209
CRP level, mg/L	68.9 (38.9 , 139.5)	62.9 (35.6 , 79.9)	110.8 (42.0 , 247.2)	0.082

Data were represented as mean ± SD and compared using two-sample *t*-test if data followed normal distribution; or median (IQR: 1st, 3rd quartiles) and compared using the Mann-Whitney U test if data didn’t follow normal distribution

*ANC* absolute neutrophil counts, *ABC* absolute band form cell counts, *ASC* absolute segment cell counts, *ALC* absolute lymphocyte counts

^*^ *p* < 0.05, indicates a significant difference between patients with and without pleural effusion

White blood cell immunophenotypes were analyzed in patients with adenovirus pneumonia with and without pleural effusion. Data from 3 patients without pleural effusion and 5 patients with pleural effusion are presented in Table 4. Patients without pleural effusion had significantly higher counts of CD4+ (*p* = 0.034), CD8+ (*p* = 0.031), and CD20+ T cells (*p* < 0.004).

**Table 4:** Immune phenotype of white blood cells of patients with adenovirus pneumonia based on the presence or absence of pleural effusion (*n* = 8)

Variables	Adenoviral pneumonia without pleural effusion (*n* = 3)	Adenoviral pneumonia with pleural effusion (*n* = 5)	*P* value
WBC count, cells/mm^3^	13500 (11500 , 18600)	6400 (5400 , 12800)	0.089
Lymphocyte counts, cells/mm^3^	2418 (1150 , 2835)	1802 (403 , 2530)	0.451
CD4^+^ T cells, cells/mm^3^	597 (465 , 1321)	130 (102 , 319)	0.034*
CD8^+^ T cells, cells/ mm^3^	601 (284 , 621)	108 (70 , 286)	0.031*
CD20^+^ T cells, cells/ mm^3^	439 (216 , 445)	35 (14 , 112)	0.004*
NK cells, cells/ mm^3^	77 (15 , 295)	23 (2 , 65)	0.393

*p < 0.05, indicates a significant difference between patients with and without pleural effusion

## Discussion

In this retrospective case series study, we analyzed the data of 27 patients with adenovirus pneumonia from the 2011 outbreak in Taiwan, and compared clinical features and laboratory data between patients with or without parapneumonic effusion using pleural effusion as a marker for disease severity. Our data showed that the presence of parapneumonic effusion was significantly associated with a longer febrile duration, more complicated clinical management, and a greater risk of extrapulmonary involvement, notably hepatitis, compared to patients without parapneumonic effusion. Patients with pleural effusion had significantly fewer leukocytes and T lymphocytes (CD4 +, CD8+, or CD20+) compared to patients without pleural effusion, suggesting that the presence of parapneumonic effusion in adenoviral pneumonia was a hallmark of depressed host cellular immunity.

It has been reported that approximately 40% of children hospitalized with pneumonia had pleural effusion [24, 25]. Pleural effusion in children is most frequently seen as a complication of bacterial pneumonia, while viral pneumonia is more frequently associated with acute respiratory distress syndrome (ARDS) than with pleural effusion. The relationship between pleural effusion and viral pneumonia has not been fully explored. The prevalence rates of pleural effusion in influenza pneumonia and RSV pneumonia are 19.1%, and 6%, respectively [26, 27]. There are limited reports regarding the prevalence of pleural effusion in adenovirus pneumonia, with estimates ranging from 37.5 to 48%. In pneumonia associated with H1N1 influenza, patients with pleural effusion had more lymphopenia, higher C-reactive protein level and more need for oxygen therapy compared to the non-effusion group. These data suggested that there was a significant difference in immunologic response between patients in the pleural effusion group and those in the non-pleural effusion group.

A total of 674 adenovirus isolates were identified during the 2011 outbreak, of which 215 cases were hospitalized. Our data identified 27 cases with severe adenovirus pneumonia. In contrast, 80% of hospitalized patients were diagnosed with mild to moderate upper respiratory tract infections. Our present results, showing that patients with pleural effusion had more severe disease compared to patients without pleural effusion, were consistent with previous studies analyzing the 2011 Taiwan outbreak, which showed that patients with severe infection had a longer duration of fever and higher incidence of LRTI involvement compared to patients with non-severe infection [19, 20]. Almost half the patients with severe infection from these and other studies exhibited LRTI symptoms such as pneumonic consolidation, pleural effusion, dyspnea, rales and wheezing [19]. Complications such as respiratory failure, acute respiratory distress syndrome (ARDS) and hypotension are also hallmarks of severe adenovirus infections [20, 28, 29]. Indeed, the presence of co-morbidities such as pleural effusion requiring drainage, respiratory distress, wheezing and gastroenteritis have been shown to be risk factors for severe adenovirus pneumonia [30, 31]. Our study showed that although there was no significant difference between the two groups in the number of days of oxygen requirement, a significantly higher number of patients with pleural effusion required oxygen compared to the group without pleural effusion.

The innate immune response has been shown to play an important role in the host response to HAdV infections [32, 33]. The initial inflammation in adenovirus pneumonia has been shown to begin with a neutrophilic interstitial infiltrate, followed by appearance of monocytes, then a lymphocytic infiltrate and release of IL-8 and IL-6 [21]. Other studies reported that intravenous injection of large doses of adenovirus caused leukopenia in non-human primates, humans and hamsters, with a significant decrease in the number of CD3+, CD4+ and CD8+ cells at 1 day post-challenge [34–36]. It has been suggested that adenovirus infections in immunocompetent individuals could result from inhibition of cytokine production, suppression of T cell function, and inhibition of major histocompatibility complex (MHC) expression by virulent strains like HAdV-3 and HAdV-7 [37]. Recombinant adenovirus 35 (rAd35) has been shown to inhibit DC-induced activation of CD4+ cells by specifically binding to CD46 on the surface of T cells and suppressing proliferation and cytokine production in memory cells as well as in total CD4+ cells [38]. Our study data suggested that the presence of parapneumonic effusion in adenoviral pneumonia was significantly associated with depression of host cellular immunity, and were consistent with previous findings that patients with severe infection more frequently had leukopenia and thrombocytopenia compared to patients with non-severe infection [19, 20]. It is important to note that in our study, lymphocyte counts were calculated only for cases where data for immune cell subpopulations were available (*n* = 5), whereas ALC numbers were calculated for all cases. The lymphocyte counts were therefore different from ALC counts. Since inflammation of the respiratory tract is typically associated with upregulation of ALCs, lymphocytes and T cells, we suggest that depression of host cellular immunity during parapneumonic effusion could be because of re-distribution of these immune cells. Our data indicated that patients without pleural effusion had significantly higher WBC counts compared to patients with pleural effusion. It will be interesting to validate our findings in a larger sample size to understand the association between disease severity and leukocytosis. Based on our data, it is likely that preventive measures for opportunistic infections may be effective when patients with adenovirus pneumonia present with parapneumonic effusion.

In addition to lung injury, severe adenovirus infection has been reported to also result in liver injury, with elevated levels of serum lactate dehydrogenase (LDH) and aspartate aminotransferase (AST) correlating with severity of adenovirus infection [20, 39]. In our present study, the incidence of hepatitis was significantly higher in patients with pleural effusion compared to patients without pleural effusion, suggesting the presence of a more severe systemic inflammatory process in these patients.

## Conclusions

To the best of our knowledge, this study is the first to analyze T-cell profiles in relation to the presence of parapneumonic effusion in patients with adenoviral pneumonia. Our data were consistent with previous findings from the 2011 community outbreak in Taiwan, and indicated that severity of adenovirus infection correlated positively with suppression of T-cell immunity. Although the mechanism remains unclear, it is likely that T-cell depression may be due to direct inhibition by adenovirus, or non-specific inhibition associated with immunoparalysis at late sepsis. Further prospective, large-scale studies are needed to further characterize the clinical outcomes and risks for subsequent infections among these patients. An important drawback of this study was the small sample size. Therefore, although it was previously reported that patients infected with HAdV-7 had a higher mortality rate in the 2011 outbreak [20], the sample size of this study precluded our ability to stratify our study patients based on their HAdV-3 or HAdV-7 status for statistical comparison. Additionally, there were no deaths over the course of our study period. However, even with limited case numbers, we found a significant difference in immune profiles between patients with simple pneumonia and those with pleural effusion. It will be interesting to evaluate dynamic changes in immune cell profiles through the course of infection to better understand the interaction between viral and immune cells.

## Abbreviations

ARDS: Acute respiratory distress syndrome
ASCs: Absolute segment cell counts
AST: Aspartate aminotransferase
DFA: Direct fluorescent-antibody assay
ECMO: Extracorporeal membrane oxygenation
HAdV: Human adenovirus
HFOV: High frequency oscillatory ventilation
LDH: Lactate dehydrogenase
LRTIs: Lower respiratory tract infections
MHC: Histocompatibility complex
NCKU: National Cheng Kung University
rAd35: Recombinant adenovirus 35
RSV: Respiratory syncytial virus

## References

[1] Lynch JP, Fishbein M, Echavarria M (2011). Adenovirus. Semin Respir Crit Care Med.

[2] Ebner K, Pinsker W, Lion T (2005). Comparative sequence analysis of the hexon gene in the entire spectrum of human adenovirus serotypes: phylogenetic, taxonomic, and clinical implications. J Virol.

[3] Russell WC (2009). Adenoviruses: update on structure and function. J Gen Virol.

[4] Edwards KM, Thompson J, Paolini J, Wright PF (1985). Adenovirus infections in young children. Pediatrics.

[5] Tang L, Wang L, Tan X, Xu W (2011). Adenovirus serotype 7 associated with a severe lower respiratory tract disease outbreak in infants in Shaanxi Province, China. Virol J.

[6] Sun B, He H, Wang Z, Qu J, Li X, Ban C (2014). Emergent severe acute respiratory distress syndrome caused by adenovirus type 55 in immunocompetent adults in 2013: a prospective observational study. Crit Care.

[7] Kim SJ, Kim K, Park SB, Hong DJ, Jhun BW (2015). Outcomes of early administration of cidofovir in non-immunocompromised patients with severe adenovirus pneumonia. PLoS One.

[8] Low SY, Tan TT, Lee CH, Loo CM, Chew HC (2013). Severe adenovirus pneumonia requiring extracorporeal membrane oxygenation support--Serotype 7 revisited. Respir Med.

[9] Hung KH, Lin LH (2015). Adenovirus pneumonia complicated with acute respiratory distress syndrome: a case report. Medicine (Baltimore).

[10] Alcorn MJ, Booth JL, Coggeshall KM, Metcalf JP (2001). Adenovirus type 7 induces interleukin-8 production via activation of extracellular regulated kinase 1/2. J Virol.

[11] Munoz FM, Piedra PA, Demmler GJ (1998). Disseminated adenovirus disease in immunocompromised and immunocompetent children. Clin Infect Dis.

[12] Alpert G, Charney E, Fee M, Plotkin SA (1986). Outbreak of fatal adenoviral type 7a respiratory disease in a children’s long-term care inpatient facility. Am J Infect Control.

[13] Klinger JR, Sanchez MP, Curtin LA, Durkin M, Matyas B (1998). Multiple cases of life-threatening adenovirus pneumonia in a mental health care center. Am J Respir Crit Care Med.

[14] Glezen P, Denny FW (1973). Epidemiology of acute lower respiratory disease in children. N Engl J Med.

[15] Gu L, Liu Z, Li X, Qu J, Guan W, Liu Y (2012). Severe community-acquired pneumonia caused by adenovirus type 11 in immunocompetent adults in Beijing. J Clin Virol.

[16] Ng OT, Thoon KC, Chua HY, Tan NW, Chong CY, Tee NW (2015). Severe pediatric adenovirus 7 disease in Singapore linked to recent outbreaks across Asia. Emerg Infect Dis.

[17] Lin KH, Lin YC, Chen HL, Ke GM, Chiang CJ, Hwang KP (2004). A two decade survey of respiratory adenovirus in Taiwan: the reemergence of adenovirus types 7 and 4. J Med Virol.

[18] Chang SY, Lee CN, Lin PH, Huang HH, Chang LY, Ko W (2008). A community-derived outbreak of adenovirus type 3 in children in Taiwan between 2004 and 2005. J Med Virol.

[19] Tsou TP, Tan BF, Chang HY, Chen WC, Huang YP, Lai CY (2012). Community outbreak of adenovirus, Taiwan, 2011. Emerg Infect Dis.

[20] Lai CY, Lee CJ, Lu CY, Lee PI, Shao PL, Wu ET (2013). Adenovirus serotype 3 and 7 infection with acute respiratory failure in children in Taiwan, 2010–2011. PLoS One.

[21] Wu W, Booth JL, Duggan ES, Patel KB, Coggeshall KM, Metcalf JP (2010). Human lung innate immune cytokine response to adenovirus type 7. J Gen Virol.

[22] Ginsberg HS, Moldawer LL, Sehgal PB, Redington M, Kilian PL, Chanock RM (1991). A mouse model for investigating the molecular pathogenesis of adenovirus pneumonia. Proc Natl Acad Sci U S A.

[23] Clark TW, Fleet DH, Wiselka MJ (2011). Severe community-acquired adenovirus pneumonia in an immunocompetent 44-year-old woman: a case report and review of the literature. J Med Case Rep.

[24] Mocelin HT, Fischer GB (2002). Parapneumonic pleural effusion. Pediatr Respir Rev.

[25] Hamm H, Light RW (1997). Parapneumonic effusion and empyema. Eur Respir J.

[26] Kern S, Uhl M, Berner R, Schwoerer T, Langer M (2001). Respiratory syncytial virus infection of the lower respiratory tract: radiological findings in 108 children. Eur Radiol.

[27] Kim YN, Cho HJ, Cho YK, Ma JS (2012). Clinical significance of pleural effusion in the new influenza A (H1N1) viral pneumonia in children and adolescent. Pediatr Pulmonol.

[28] Wenman WM, Pagtakhan RD, Reed MH, Chernick V, Albritton W (1982). Adenovirus bronchiolitis in Manitoba: epidemiologic, clinical, and radiologic features. Chest.

[29] Hong JY, Lee HJ, Piedra PA, Choi EH, Park KH, Koh YY (2001). Lower respiratory tract infections due to adenovirus in hospitalized Korean children: epidemiology, clinical features, and prognosis. Clin Infect Dis.

[30] Rajkumar V, Chiang CS, Low JM, Cui L, Lin RT, Tee NW (2015). Risk factors for severe adenovirus infection in children during an outbreak in Singapore. Ann Acad Med Singapore.

[31] Gupta P, Goyal S, Tobias JD, Prodhan P, Purohit P, Gossett JM (2011). Risk factors associated with hospital admission among healthy children with adenovirus infection. Turk J Pediatr.

[32] Hendrickx R, Stichling N, Koelen J, Kuryk L, Lipiec A, Greber UF (2014). Innate immunity to adenovirus. Hum Gene Ther.

[33] Schagen FH, Ossevoort M, Toes RE, Hoeben RC (2004). Immune responses against adenoviral vectors and their transgene products: a review of strategies for evasion. Crit Rev Oncol Hematol.

[34] Schnell MA, Zhang Y, Tazelaar J, Gao GP, Yu QC, Qian R (2001). Activation of innate immunity in nonhuman primates following intraportal administration of adenoviral vectors. Mol Ther.

[35] Freytag SO, Stricker H, Movsas B, Kim JH (2007). Prostate cancer gene therapy clinical trials. Mol Ther.

[36] Toth K, Lee SR, Ying B, Spencer JF, Tollefson AE, Sagartz JE (2015). STAT2 knockout Syrian hamsters support enhanced replication and pathogenicity of human adenovirus, revealing an important role of type I interferon response in viral control. PLoS Pathog.

[37] Hakim FA, Tleyjeh IM (2008). Severe adenovirus pneumonia in immunocompetent adults: a case report and review of the literature. Eur J Clin Microbiol Infect Dis.

[38] Adams WC, Gujer C, McInerney G, Gall JG, Petrovas C, Karlsson Hedestam GB (2011). Adenovirus type-35 vectors block human CD4+ T-cell activation via CD46 ligation. Proc Natl Acad Sci U S A.

[39] Spaeder MC, Fackler JC (2011). Hospital-acquired viral infection increases mortality in children with severe viral respiratory infection. Pediatr Crit Care Med.

